# Smooth Return to Work Through Early Rehabilitation and Interdisciplinary Collaboration After Ostomy: A Case Report of a Japanese Patient

**DOI:** 10.7759/cureus.65052

**Published:** 2024-07-21

**Authors:** Shinno Iijima

**Affiliations:** 1 Medical Technology Department, Rehabilitation Office, International University of Health and Welfare Hospital, Nasushiobara, JPN

**Keywords:** postoperative care, artificial anus, stoma, occupational therapy, physical therapy rehabilitation, multidisciplinary collaboration, early rehabilitation, colostomy patients

## Abstract

A male patient in his 40s was diagnosed with rectal cancer and underwent abdominoperineal resection (APR) with permanent end colostomy as surgical treatment. He wanted to return to work as soon as possible after discharge. A physical therapist (PT) was involved in the preoperative consultation, and both the PT and occupational therapist started bed rest and activities of daily living (ADL) practice the day after surgery. On the third postoperative day, lightweight trunk exercises were initiated with a gradual increase in load. Stoma management was supervised by a nurse and progress was monitored. The patient’s progress in ADLs, postoperative complications, and return to work were evaluated two weeks after discharge. Consequently, the patient was able to continue rehabilitation without early complications related to postoperative stoma. He could lift 20 kg and return to carpentry two weeks after discharge. The stoma quality of life improved from 61 points at two weeks after surgery to 74 points at two weeks after discharge. Early rehabilitation for social reintegration after ostomy creation can be safely performed under PT supervision, and a comprehensive interprofessional collaboration can contribute to smooth social reintegration.

## Introduction

An artificial anus (colostomy) is occasionally used as a surgical treatment for colon and rectal cancers. Unlike the traditional anus, an ostomy creates a new anal passage in the abdomen. There are two types of stomas: those created temporarily for therapeutic reasons (cases that will undergo closure surgery in the future), and permanent stomas that are used for the rest of the patient's life. It has been reported that patients with permanent ostomies tend to have reduced activities of daily living (ADL) and quality of life (QoL) due to changes in management and body image [1,2]. The presence of a gastrointestinal stoma, especially in patients of working age, can sometimes be an obstacle when returning to work [3,4]. 

The Association of Stoma Care Nurses United Kingdom (ASCN UK) clinical guidelines recommend support from healthcare providers, including exercise [5]; however, there are currently no systematic protocols for assessing the exercise content required for rehabilitation. In addition, a model for multidisciplinary collaboration has not yet been established. Therefore, it is necessary to report on efforts made in the clinical field and verify the effectiveness of medical support.

In this study, we had the opportunity to perform perioperative rehabilitation on a patient with rectal cancer who underwent a colostomy (stoma) after surgery. By introducing exercises focusing on post-discharge activities from the early postoperative period, the physical therapist (PT) collaborated with the occupational therapist (OT), nurse, and physicians, leading to a smooth return to work.

This article was previously presented as a meeting abstract at the 27th Annual Scientific Meeting of the Tochigi Physical Therapy Association on November 26, 2023.

## Case presentation

The patient was a 40-year-old male. He lived with his wife and worked as a professional carpenter. His body mass index (BMI) was 20.3 kg/m², which is within the normal range for Japanese individuals. There was no observed weight loss over the past three months, suggesting that the likelihood of cancer cachexia, or sarcopenia was low. He smoked in his 20s but quit in his 30s and was an occasional drinker. He had no prior medical history or complications that required treatment. He presented to the hospital after undergoing health screening in his community, indicating the need for evaluation. He was diagnosed with stage II rectal cancer. The patient's Eastern Cooperative Oncology Group (ECOG) Performance Status score was 0, indicating that he was able to perform ADLs without any limitations post-onset. The patient was indicated for radical surgical treatment. Neoadjuvant chemoradiotherapy was not performed in this case. The patient underwent abdominoperineal resection (APR) with permanent end colostomy on day Z. During the perineal procedure, the perineal body between the prostate and rectum was resected, followed by suturing of the fascia and skin, and a drain was placed. After the surgery, the patient was discharged from the hospital on postoperative day Z+27 and had to return to work as soon as possible. The patient had to be able to use his tools while standing and lift 20 kg of wood. The postoperative process of rehabilitation and return to work is given in detail below.

Progress and examination items

Table 1 shows the progress in ADL, postoperative complications, and rehabilitation from the time of hospital admission until returning to work. The patient was admitted to the hospital two days before surgery and received preoperative care and education from the medical staff. He had no pain during the preoperative period, with a score of 0 on the Numerical Rating Scale (NRS). He had no preoperative complications and showed no signs of sarcopenia or cachexia. Additionally, no severe postoperative complications, such as hemodynamic changes or pneumonia, were observed. Therefore, after surgery, early postoperative mobilization was permitted by the physician following the globally recognized Enhanced Recovery After Surgery (ERAS) protocol. Rehabilitation was initiated the next day, in addition to the usual nursing care and stoma care. On day Z+13, he required medical intervention for the perineal wound due to a foul odor and purulent discharge. This necessitated irrigation, medication, and close monitoring. He was discharged on Z+27 days after wound management was completed, he was able to use the pouch technique, and could independently perform ADLs. In this case, no chemotherapy was administered after the surgery.

**Table 1:** Progress from hospitalization to return to work and the involvement of each medical profession. Z: day of surgery; PT: physical therapy; OT: occupational therapy; NS: nursing; SLR: straight leg raising

Day	Z-2	Z-1	Z	Z+1	Z+7	Z+13	Z+16	Z+21	Z+27	Z+34	Z+41
-	Hospitalization	Informed consent	Surgery	Assessment of general condition	Remove stitches	Wound eruption＋	-	Wound eruption(–)	Discharge	Follow-up	Return to work
PT	-	Breathing exercises Preoperative evaluation		Respiratory rehabilitation Early ambulation	Draw-in exercise (supine) SLR exercise	Draw-in exercise (sit) Stair climbing	Squatting Outdoor walking	Lift a 2~5 kg weight from waist height	Lift a 7~12 kg weight from waist height	Lift a 20 kg weight from waist height	Final assessment
OT	-	-		Early ambulation Seat adjustment	Upper limb exercises Seated exercises	Upper limb exercises Seated exercises	Tool use training (sit)	Tool use training (stand)	Tool use training (squatting position)	Checking activities of daily living	Final assessment
NS	Preoperative evaluation Stoma site marking			Wound care	Wound care, stoma care, and pouch change instruction	Wound care, stoma care, and pouch change instruction				Stoma care follow up	

Perioperative physical function and quality of life were assessed by the PT, which included grip strength, a six-minute walk test (6MWT), and the Japanese Society of Wound Ostomy and Continence Management (JWOCM) Stoma-QoL questionnaire. The Japanese version of the Stoma-QOL was created by JWOCM and is publicly available (free for members). It is widely used as an evaluation tool within Japan. The Stoma-QoL is a four-item self-administered questionnaire consisting of 20 questions (maximum score 80 points) related to pouch problems, outings, changes in body image, and relationships with others. These evaluation items were selected to assess muscle strength, endurance, and the QoL from the patient's perspective. The perioperative blood data, changes in physical function, and changes in QoL are shown in Table 2.

**Table 2:** Blood tests, pain, physical function, and quality of life results. Z: day of surgery; NRS: Numerical Rating Scale; 6MWT: 6-Minute Walk Test, Stoma-QoL: Stoma Quality of Life questionnaire (Japan)

	Baseline	Postoperative days	
	Z-1	Z+13	Z+41
Albumin (g/dl)	3.4	2.9	3.2
C-reactive protein (mg/dl)	0.03	3.44	0.22
Hemoglobin (g/dl)	13.3	12.2	11.9
NRS during activity (at rest)	0 (0)	3-4 (1)	0 (0)
Grip strength (Right/Left [kg])	48.6/48.0	41.0/45.0	51.2/52.1
6MWT (m)	520	400	510
Stoma-QoL score	-	61	74

Rehabilitation

On the day of admission, the PT initiated rehabilitation at the request of the attending physician. Respiratory instructions were provided to prevent pneumonia [6], and informed consent was obtained for planned postoperative rehabilitation. Preoperative physical function was assessed at baseline. His grip strength was 48.6/48.0 kg, and the 6MWT distance was 520 m. Stoma-QoL was assessed in the second postoperative week and at return to work. On the day after surgery, the PT and OT began weaning him from the bed, to avoid abdominal muscle contraction, and practicing the ADLs. From the first postoperative week, under the supervision of the PT, he was placed in the supine, sitting, standing, and lifting positions to gradually increase the load on the abdominal muscles. Load gradation was determined with reference to a study that measured abdominal pressure during postures and movements in healthy subjects [7].

**Figure 1:**
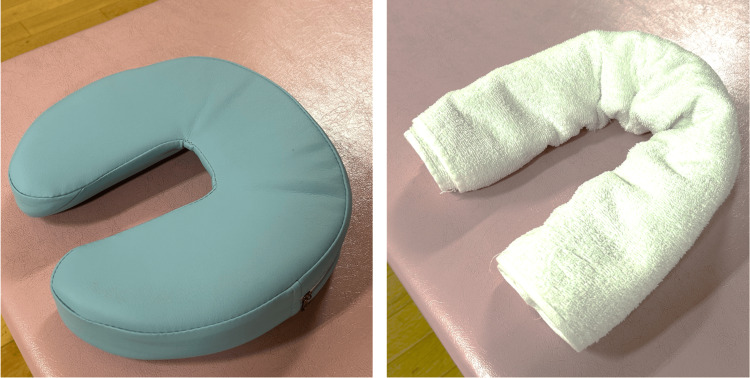
Use of cushions and towels to alleviate incisional pain in a seated position.

Physical function was evaluated at two weeks postoperatively; grip strength was 41.0/45.0 kg, the 6MWT distance was 380 m, and the Stoma-QoL score was 61. His NRS was 1 at rest, 3-4 when sitting and walking. The improvement in physical function was judged to be good, and light-lifting activities were started approximately three weeks postoperatively. Occupational therapy was performed to strengthen the upper limb muscles based on his general condition, to improve ADLs, and to facilitate his return to work. In addition, his wound from APR with permanent end colostomy was slightly painful because of the pressure in the sitting position. To alleviate pain, we adjusted the seating surface using cushions and towels. Specifically, the cushion was adjusted to have a gap in front of the center of the perineum (U-shaped) to facilitate the load on the sciatic area in the sitting position(Figure 1).

After discharge from the hospital on Z+27, the patient continued outpatient rehabilitation. On Z+34, the patient was allowed to return to work after outpatient rehabilitation, during which it was confirmed that he could safely lift a 20 kg weight. At this stage, he experienced no pain at rest or during movement, with an NRS score of 0. As the final assessment on Z+41, physical function was evaluated and grip strength was 51.2/52.1 kg, the 6MWT distance was 510 m, and the Stoma-QoL score was 74. Specific sub-scores showed improvement in concerns about ostomy noise, odor, and fear of human interaction.

Nursing care

Preoperatively, in addition to usual care, the patient was given a preliminary explanation of stoma management and stoma site marking. Stoma site marking is the process of marking the location of a stoma on the patient's abdomen in advance. This allows the stoma to be planned such that it does not interfere with ADLs by avoiding clothing seams, skin folds, and bony prominences. Appropriately positioned ostomies facilitate ostomy management and reduce the risk of skin breakdown, contributing to an improved QOL [8]. Postoperative wound management of the stoma and perineum was performed by a physician. The patient had acquired the skills necessary for stoma management by the third postoperative week.

Interdepartmental and patient information sharing

The patient's postoperative wound and general condition were communicated to the physician, and the safety of exercise and occupational therapy was confirmed. In addition to the physician, the nurse reviewed the patient's progress in learning the colostomy pouch management technique and discussed a return-to-work policy. The PT and OT shared information with the patient on how to adjust the load on the entire body and abdominal muscle groups, sitting posture, and movements required for work. In addition, a meeting was held between the patient and his/her supervisor immediately after discharge from the hospital to share information with the workplace. During the interview, the patient's treatment and rehabilitation status were confirmed, and the nature of his work after returning to work was determined. The main considerations were as follows: (i) The patient would start with light duties for half a day upon returning to work; (ii) Assistance would be provided for lifting objects over 20 kg; (iii) The patient's physical condition and limitations would be communicated to colleagues as far as the patient permitted; (iv) Any issues encountered while performing duties would be addressed by seeking advice from healthcare professionals. The patient commented, "I was able to imagine how I will work when I return to work through the rehabilitation program that simulates work" and "I will be able to return to society with less resistance than I imagined during my hospitalization" since the specific work style was discussed with the workplace. The patient did not complain of any physical or psychological pain, including wounding upon returning to work.

## Discussion

Regarding early postoperative rehabilitation during hospitalization, improving physical activity from an early stage is expected to improve physical function, shorten the hospital stay, and improve QoL in patients with rectal and colorectal cancer [9,10]. It has been suggested that continuing interventions to increase activity not only during hospitalization but also after discharge may increase the rate of return to work [11]. Additionally, a survey of the predictors of early return to work reported that low fatigue, high work value, high work capacity, and high occupational self-efficacy facilitated return to work [12]. Therefore, we believe that it is important to reacquire work capacity while increasing motor endurance as part of the rehabilitation program. In this case, early rehabilitation for social reintegration was provided under the supervision of a PT, and the amount of activity was safely increased. The patient required 27 days postoperatively until discharge due to perineal wound management. However, if there had been no issues with the wound, earlier discharge and return to work might have been possible.

Although avoiding excessive abdominal pressure in the early postoperative period is a clinical principle for preventing early complications after stoma creation, there are no reports that provide specific protocols. We referred to a report on bladder pressure measurement in healthy subjects during the progression of rehabilitation. It is known that intra-abdominal pressure increases with changes in posture and movement, such as walking on flat ground or climbing stairs. For example, when coughing is involved, intra-abdominal pressure is generally lower in a sitting position compared to standing. The order of movements performed was judged to be less likely to cause abdominal pressure, thereby contributing to increased safety [7].

In addition, since APR with permanent end colostomy tends to cause pain in the sitting position, we discussed strategies to make the "sitting position" comfortable with the OT, which we felt was a good point to promote the patient's bed release in this case. Once a patient is in a general condition that allows stable bed release, it is important to understand the patient's unique work requirements and incorporate them into the rehabilitation program. The National Institute for Health and Care Excellence clinical guidelines advocate the importance of an individualized rehabilitation plan based on the patient’s unique needs and goals [13]. The results suggest that comprehensive patient-centered interprofessional collaboration, such as occupational rehabilitation with an OT and PT, during the postoperative course to meet the patient's needs, and a guidance system for pouch management by the nurse may facilitate smooth reintegration into society.

In terms of environmental and social support, the patient had good opportunities to discuss tasks with his workplace based on his understanding of the tasks that he was currently capable of performing. The fact that the patient and his supervisor were able to share an image of his smooth return to work and that the workplace accepted the patient well may have contributed to the improvement in the QoL index. In this case, the patient communicated well with the workplace; however, in other cases, it may be necessary to consider the role of medical staff as an intermediary to provide explanations and advice. In cases where support for returning to work is difficult, connecting the patient not only to medical staff but also to a specialized coordinator is also a matter to consider [14].

A limitation of this case was that specific values predicting the abdominal load could not be evaluated. It would be safer if an evaluation using a numerical value as an indicator could be performed in parallel with the exercise. For example, the increase in load could have been predicted in real time by measuring the thickness of the trunk muscles with an ultrasound device or by measuring the increase or decrease in trunk muscle activity with a surface electromyograph. Because a measurement environment was not available in this case, we will consider this as a future issue. Safety protocols for patients with ostomies in the acute to chronic phase have not yet been established worldwide. In addition to acute complications, parastomal hernia is a representative long-term complication. This condition is caused by factors such as weakening of the abdominal wall, obesity, and increased intra-abdominal pressure due to constipation [15]. It is necessary to investigate whether the provision of exercise therapy by physical therapists can help prevent such complications.

Through this case, we learned that the development of a structured protocol involving multiple professionals is essential for patients to reintegrate into society and improve their QoL after stoma creation. As a perspective for future research, longitudinal studies are needed to investigate the impact of early rehabilitation and interdisciplinary collaboration on patients' QoL and social reintegration.

## Conclusions

A comprehensive interdisciplinary approach and early rehabilitation contributed to the patient's successful return to work and improved QoL. This case highlights the importance of an individualized rehabilitation plan, effective interdisciplinary collaboration, and a supportive work environment in the management of patients with ostomies. Further research and the development of structured care models are essential to improve patient outcomes and support reintegration.
